# Resveratrol Protects Rat Ovarian Luteinized Granulosa Cells from H_2_O_2_-Induced Dysfunction by Activating Autophagy

**DOI:** 10.3390/ijms241310914

**Published:** 2023-06-30

**Authors:** Minghui Cai, Haijuan Sun, Yujia Huang, Haixu Yao, Chen Zhao, Jiao Wang, Hui Zhu

**Affiliations:** Department of Physiology, Harbin Medical University, Harbin 150081, China; caiminghui@hrbmu.edu.cn (M.C.); sunhj0922@163.com (H.S.); 102404@hrbmu.edu.cn (Y.H.); haixuyao2022@163.com (H.Y.); zczczcii@163.com (C.Z.); 200135@hrbmu.edu.cn (J.W.)

**Keywords:** autophagy, resveratrol, corpus luteum, luteinized granulosa cell

## Abstract

Resveratrol performs a variety of biological activities, including the potential regulation of autophagy. However, it is unclear whether resveratrol protects against luteal dysfunction and whether autophagy involves the regulation of resveratrol. This study aims to investigate whether resveratrol can regulate autophagy to resist H_2_O_2_-induced luteinized granulosa cell dysfunction in vitro. Our results showed that resveratrol can enhance cell viability, stimulate the secretion of progesterone and estradiol, and resist cell apoptosis in H_2_O_2_-induced luteinized granulosa cell dysfunction. Resveratrol can activate autophagy by stimulating the expression of autophagy-related genes at the transcriptional and translational levels and increasing the formation of autophagosomes and autophagolysosomes. Rapamycin, 3-methyladenine, and bafilomycin A1 regulated the levels of autophagy-related genes in H_2_O_2_-induced luteinized granulosa cell dysfunction and further confirmed the protective role of autophagy activated by resveratrol. In conclusion, resveratrol activates autophagy to resist H_2_O_2_-induced oxidative dysfunction, which is crucial for stabilizing the secretory function of luteinized granulosa cells and inhibiting apoptosis. This study may contribute to revealing the protective effects of resveratrol on resisting luteal dysfunction from the perspective of regulating autophagy.

## 1. Introduction

The corpus luteum (CL) is a temporary and important internal structure in the ovary that evolves from a remnant follicle after ovulation with a main function of the synthesis of steroid hormones such as progesterone, estradiol, and androstenedione [1,2]. The evolution of the corpus luteum includes development, maintenance, and degradation, which are essential for maintaining a normal menstrual cycle, regulating the development of follicles, promoting the implantation of embryos, and protecting the safety of early pregnancy [3,4]. Dysfunction of the corpus luteum is involved in some diseases of the corpus luteum such as corpus luteum rupture and luteal phase deficiency [5,6].

Oxidative stress is one of the main causes of luteal dysfunction, particularly the metabolic production of superoxide and hydrogen peroxide (H_2_O_2_), which are common inducers [7]. These excessive reactive oxygen species accelerate luteal dysfunction by directly attacking proteins, lipids, nucleic acids, and other biological macromolecules and indirectly activating abnormal aging and apoptosis signals as second messengers [8,9]. H_2_O_2_ has a broad impact on mitochondrial dysfunction, DNA mutation, accelerating aging, initiating apoptosis, and other pathological progressions [10,11,12]. However, effective preventative measures and treatments for luteal dysfunction induced by H_2_O_2_ are still lacking.

Autophagy is a unique adaptive reaction of eukaryotic cells. Various substances or organelles can be degraded and reabsorbed by autophagy to meet the survival needs of cells. Autophagy goes through four key stages: the formation of preautophagosomal structures stimulated by autophagy initiation factors, phagophore wrapping of substrates to form autophagosomes, autophagosome and lysosome fusion to form autophagolysosomes, and degradation of substrates in autolysosomes [13]. Autophagy plays an important role in a variety of pathological conditions, such as hypoxia, starvation, inflammation, and oxidative stress, but its effects remain controversial [14]. Liu et al. found that autophagy played a neuroprotective role in spiral ganglion neurons treated with cisplatin both in vitro and in vivo, indicating that apoptosis and related hearing loss were alleviated after autophagy activation [15]. However, inhibition of autophagy with 3-methyladenine (3-MA), LY294002, or Atg7 silencing prevented the expression of senescence-associated markers in serum-starved fibroblasts to achieve protective effects [16]. Zhang et al. also found that autophagy activation exacerbated cytotoxicity and apoptosis, while autophagy inhibition ameliorated H_2_O_2_-mediated granulosa cell apoptosis [17]. Based on these current studies, the role of autophagy in various pathological processes remains to be further revealed.

Resveratrol (RSV) is a natural polyphenolic compound widely found in the fruits, roots, and stems of grapes, mulberries, peanuts, and other plants with a variety of biological functions, such as anticancer, anti-inflammation, neuroprotection, and cardioprotection [18,19]. Some studies have focused on the regulation of autophagy by resveratrol in the ovaries, but the conclusions are still uncertain. For instance, Yong et al. found that resveratrol attenuated the effects of malathion on inducing autophagy and apoptosis in granulosa cells [20]. Jiao et al. found that resveratrol inhibited autophagy by reducing BPA-induced autophagic vesicle formation in granulosa cells [21]. However, resveratrol was also found to activate autophagy and mitochondrial biogenesis in oocytes and granulosa cell complexes [22]. These results suggest that further exploration of the regulatory effect of resveratrol on autophagy in the ovaries is necessary.

Based on these previous studies, the effects of resveratrol on luteal dysfunction might be associated with autophagy. Thus, we detected the effects of resveratrol on H_2_O_2_-induced luteinized granulosa cell dysfunction, autophagy morphological changes, and the levels of autophagy-related genes. This study aims to demonstrate whether resveratrol protects luteinized granulosa cells from H_2_O_2_-induced oxidative dysfunction by regulating the autophagy level.

## 2. Results

### 2.1. Resveratrol Protected H_2_O_2_-Induced Luteinized Granulosa Cell Dysfunction

**Figure 1:**
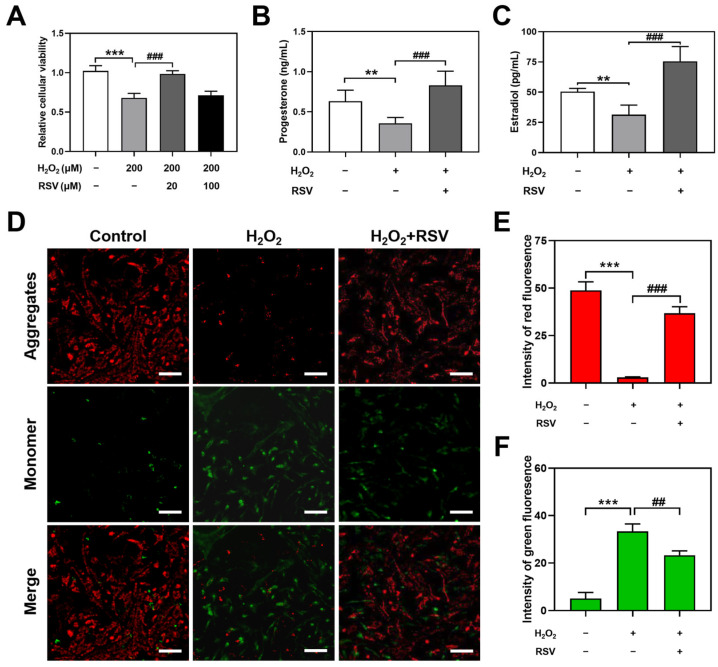
Resveratrol protected H_2_O_2_-induced luteinized granulosa cell dysfunction. (**A**) The relative cellular viability was detected using CCK-8 assay (*n* = 5). (**B**,**C**) The secretion of progesterone and estradiol was determined using radioimmunoassay (*n* = 4). (**D**) Early apoptosis was detected using JC-1 staining. Scale bar: 50 µm. (**E**,**F**) Quantitative analysis of mean fluorescence intensity (*n* = 3). Compared with the control group, ** *p* < 0.01, *** *p* < 0.001; compared with the H_2_O_2_ group, ## *p* < 0.01, ### *p* < 0.001.

To observe the effects of resveratrol on H_2_O_2_-induced luteinized granulosa cell dysfunction, 200 μM H_2_O_2_-induced luteinized granulosa cells were cotreated with 20 μM or 100 μM resveratrol for 24 h, and the concentration of H_2_O_2_ was described in a previous study [23]. Compared with the control group, 200 μM H_2_O_2_ treatment significantly decreased the relative cellular viability (*p* < 0.001), while 20 μM resveratrol significantly reversed H_2_O_2_-induced luteinized granulosa cell dysfunction (*p* < 0.001), which was used as an appropriate protective condition for subsequent studies (Figure 1A). However, a significant difference in cellular viability was not observed between the 200 μM H_2_O_2_ group and the 200 μM H_2_O_2_ + 100 μM resveratrol group.

To investigate the influence of resveratrol on the secretion of progesterone and estradiol in H_2_O_2_-induced luteinized granulosa cells, a radioimmunoassay was used to assess the levels of hormone secretion in the luteinized granulosa cell culture supernatant. The results showed decreased levels of progesterone (*p* < 0.01) and estradiol (*p* < 0.01) in the H_2_O_2_ group. Compared with the H_2_O_2_ group, increased levels of progesterone (*p* < 0.001) and estradiol (*p* < 0.001) were observed in the H_2_O_2_ + RSV group (Figure 1B,C).

To further assess the resistance of resveratrol to H_2_O_2_, apoptosis was detected by using JC-1 staining, which dyed normal cells red and apoptotic cells green. Compared with the control group, the H_2_O_2_ group apparently decreased the intensity of red fluorescence (*p* < 0.001) and increased the intensity of green fluorescence (*p* < 0.001), representing a high level of apoptosis. Resveratrol significantly inhibited apoptosis in H_2_O_2_-induced luteinized granulosa cells, manifested as an increased intensity of red fluorescence (*p* < 0.001) and a decreased intensity of green fluorescence (*p* < 0.01, Figure 1D,F).

These results verified that resveratrol protected against H_2_O_2_-induced luteinized granulosa cell dysfunction involving relative cellular viability, secretion of progesterone and estradiol, and apoptosis.

### 2.2. Resveratrol Enhanced Autophagy in H_2_O_2_-Induced Luteinized Granulosa Cells

**Figure 2:**
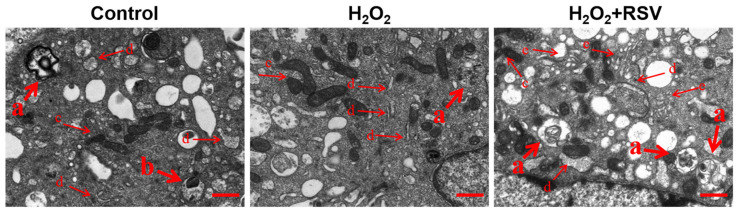
Resveratrol enhanced autophagy in H_2_O_2_-induced luteinized granulosa cells. Luteinized granulosa cells were treated with 200 μM H_2_O_2_ and 20 μM resveratrol for 24 h. The formation of autophagy was observed by transmission electron microscopy. Scale bar: 2 µm. a: autophagolysosome; b: autophagosome; c: mitochondria; d: endoplasmic reticulum; e: Golgi apparatus.

To determine the effects of resveratrol on the level of autophagy, the formation of autophagy was observed by transmission electron microscopy. The results showed that compared with the control group, there was less formation of autophagolysosomes (marked as a) and/or autophagosomes (marked as b) in the H_2_O_2_ group. However, resveratrol enhanced the formation of autophagolysosomes and/or autophagosomes in the H_2_O_2_ + RSV group, indicating that resveratrol enhanced autophagy in H_2_O_2_-induced luteinized granulosa cells (Figure 2).

### 2.3. Resveratrol Activated Autophagy by Upregulating the Levels of Autophagy-Related Genes

**Figure 3:**
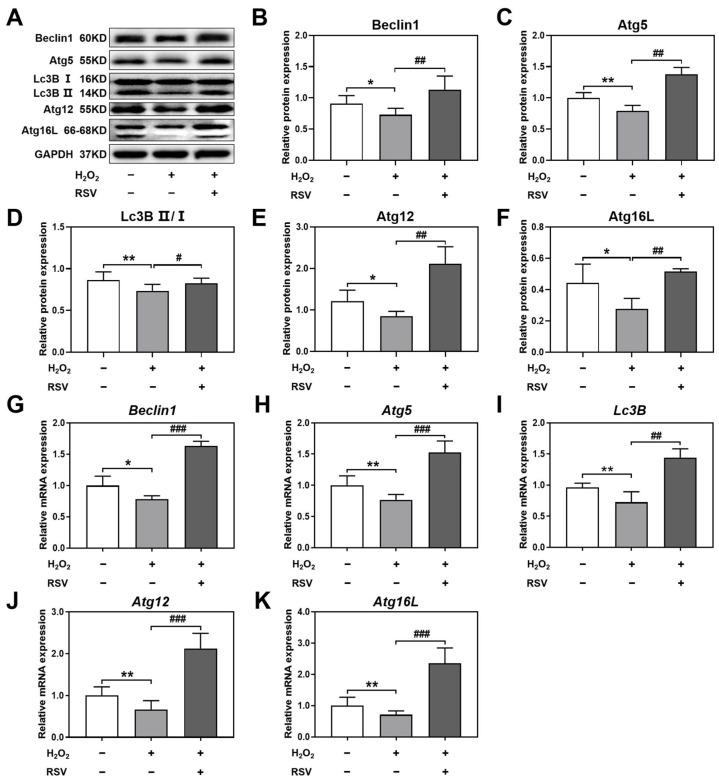
Resveratrol activated autophagy by upregulating the levels of autophagy-related genes. Luteinized granulosa cells were treated with 200 μM H_2_O_2_ and 20 μM resveratrol for 24 h. (**A**–**F**) The protein expression levels of Beclin1, Atg5, Lc3B II/I, Atg12, and Atg16L were detected using Western blotting and normalized to GAPDH (*n* = 3). (**G**–**K**) The mRNA expression levels of *Beclin1*, *Atg5*, *Lc3B*, *Atg12*, and *Atg16L* were detected using real-time PCR (*n* = 3). Compared with the control group, * *p* < 0.05, ** *p* < 0.01; compared with the H_2_O_2_ group, # *p* < 0.05, ## *p* < 0.01, ### *p* < 0.001.

To investigate the mechanism of autophagy activation induced by resveratrol, the protein levels of autophagy-related genes Beclin1, Atg5, Lc3B, Atg12, and Atg16L were detected using Western blotting. As the results show, compared with the control group, the protein expression levels of Beclin1, Atg5, Lc3B II/I, Atg12, and Atg16L in the H_2_O_2_ group were decreased significantly, which demonstrated that H_2_O_2_ induced a decline in autophagy functions in luteinized granulosa cells. However, resveratrol treatment significantly increased the protein expression levels of Beclin1, Atg5, Lc3B II/I, Atg12, and Atg16L in the H_2_O_2_ + RSV group (Figure 3A–F).

To further confirm the mRNA expression of autophagy-related genes in luteinized granulosa cells, the mRNA levels of *Beclin1*, *Atg5*, *Lc3B*, *Atg12*, and *Atg16L* were analyzed by using real-time PCR. The results showed that the mRNA levels of *Beclin1*, *Atg5*, *Lc3B*, *Atg12*, and *Atg16L* were significantly lower in the H_2_O_2_ group than in the control group, while, after treatment with resveratrol, the mRNA levels of these autophagy-related genes increased significantly, suggesting that resveratrol activated the mRNA expression of autophagy-related genes (Figure 3G–K).

These results indicated that resveratrol activated autophagy by upregulating the protein and mRNA levels of autophagy-related genes in H_2_O_2_-induced luteinized granulosa cells.

### 2.4. Autophagy Exerted Protective Effects on H_2_O_2_-Induced Luteinized Granulosa Cell Dysfunction

**Figure 4:**
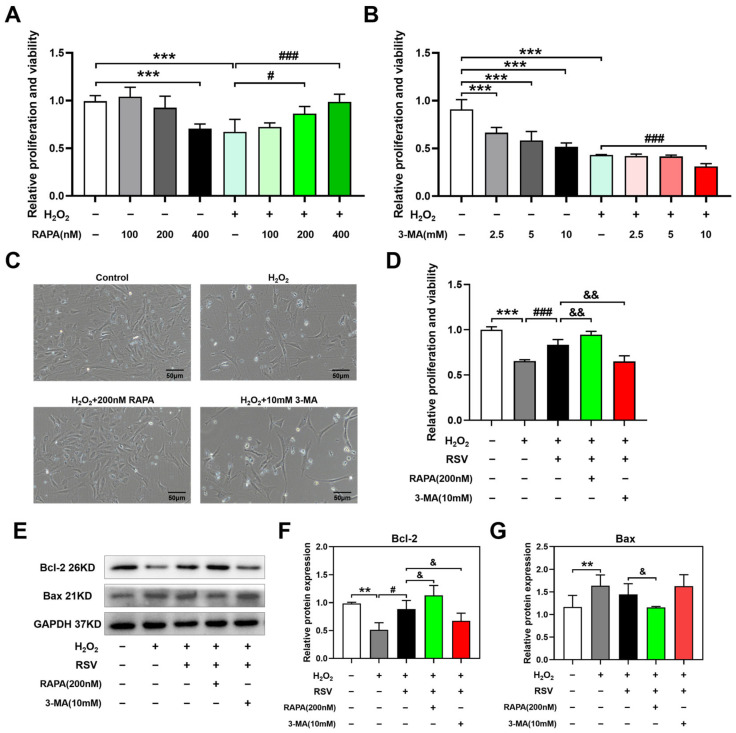
Autophagy exerted protective effects on H_2_O_2_-induced luteinized granulosa cell dysfunction. Rapamycin and 3-MA were used to treat luteinized granulosa cells for 24 h with or without 200 μM H_2_O_2_ and 20 μM resveratrol. (**A**,**B**) Relative proliferation and viability influenced by rapamycin and 3-MA (*n* = 5). (**C**) Morphological changes were observed under a phase-contrast microscope. Scale bar: 50 µm. (**D**) Relative proliferation and viability influenced by resveratrol and autophagy regulators (*n* = 5). (**E**–**G**) The levels of the apoptotic proteins Bcl-2 and Bax were determined by Western blotting (*n* = 3). Compared with the control group, ** *p* < 0.01, *** *p* < 0.001; compared with the H_2_O_2_ group, # *p* < 0.05, ### *p* < 0.001; compared with the H_2_O_2_ + RSV group, & *p* < 0.05, && *p* < 0.01.

To determine the effects of autophagy on H_2_O_2_-induced luteinized granulosa cell dysfunction, the autophagy agonist rapamycin was administered to luteinized granulosa cells to investigate the influences on relative proliferation and viability via CCK-8 assays. Compared with the control group, treatment with 100 nM and 200 nM rapamycin had no significant differences in relative proliferation and viability, while the treatment with 400 nM rapamycin caused decreased cell viability (*p* < 0.001). Compared with the H_2_O_2_ group, significantly increased cell viability was observed in the H_2_O_2_ + 200 nM rapamycin group (*p* < 0.05) and the H_2_O_2_ + 400 nM rapamycin group (*p* < 0.001, Figure 4A). Morphological results also showed more living cells and better cell morphology in the H_2_O_2_ + 200 nM rapamycin group than in the H_2_O_2_ group (Figure 4C).

To further evaluate the effects of the autophagy inhibitor 3-MA on H_2_O_2_-induced luteinized granulosa cell dysfunction, the levels of relative proliferation and viability were detected using CCK-8 assays. Compared with the control group, 2.5 mM, 5 mM, and 10 mM 3-MA induced decreased cell viability. Compared with the H_2_O_2_ group, significantly decreased cell viability was observed in the H_2_O_2_ + 10 mM 3-MA group (*p* < 0.001, Figure 4B). However, no significant morphological differences were observed between the H_2_O_2_ group and the H_2_O_2_ + 10 mM 3-MA group (Figure 4C).

To further investigate the effects of autophagy on the protective effect of resveratrol on H_2_O_2_-induced luteinized granulosa cell dysfunction, relative proliferation and viability and the levels of the apoptotic proteins Bcl-2 and Bax were assessed using CCK-8 assays and Western blotting, respectively. Compared with the H_2_O_2_ + RSV group, cotreatment with rapamycin significantly increased the relative proliferation and viability (*p* < 0.01), while cotreatment with 3-MA significantly decreased the relative proliferation and viability (*p* < 0.01, Figure 4D). Additionally, as the Western blot results showed, 200 nM rapamycin treatment upregulated the level of the anti-apoptotic protein Bcl-2 (*p* < 0.05) and downregulated the level of the pro-apoptotic protein Bax (*p* < 0.05), and 10 mM 3-MA treatment downregulated the level of the anti-apoptotic protein Bcl-2 (*p* < 0.05, Figure 4E–G).

Based on these results, autophagy regulated by rapamycin and 3-MA could further affect the protective effects of resveratrol on cell proliferation and activity and cell apoptosis in H_2_O_2_-induced luteinized granulosa cell dysfunction.

### 2.5. Rapamycin and 3-MA Regulated Autophagy in H_2_O_2_-Induced Luteinized Granulosa Cell Dysfunction

**Figure 5:**
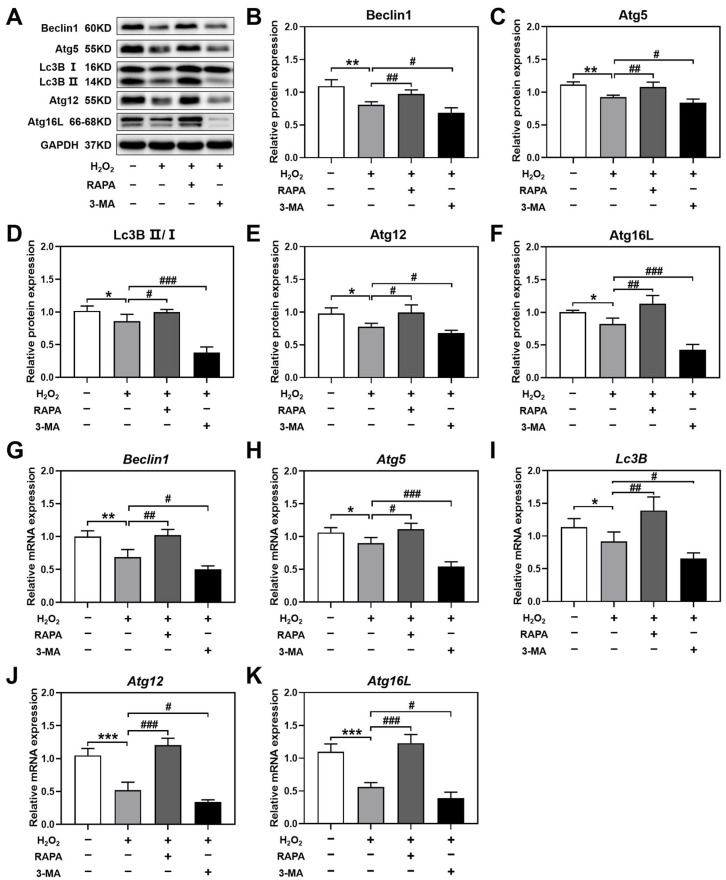
Rapamycin and 3-MA regulated autophagy in H_2_O_2_-induced luteinized granulosa cell dysfunction. Luteinized granulosa cells were treated with 200 nM rapamycin and 10 mM 3-MA and 200 μM H_2_O_2_ for 24 h. (**A**–**F**) The effects of rapamycin and 3-MA on the protein expression levels of Beclin1, Atg5, Lc3B II/I, Atg12, and Atg16L (*n* = 3). The relative protein expression normalized to GAPDH was quantified using ImageJ software version 1.46r. (**G**–**K**) The effects of rapamycin and 3-MA on the mRNA expression levels of *Beclin1*, *Atg5*, *Lc3B*, *Atg12*, and *Atg16L* (*n* = 3). Compared with the control group, * *p* < 0.05, ** *p* < 0.01, *** *p* < 0.001; compared with the H_2_O_2_ group, # *p* < 0.05, ## *p* < 0.01, ### *p* < 0.001.

To verify whether the effects of rapamycin and 3-MA on H_2_O_2_-induced luteinized granulosa cell dysfunction were dependent on the regulation of autophagy, the protein and mRNA levels of Beclin1, Atg5, Lc3B, Atg12, and Atg16L were detected using Western blotting and real-time PCR. Luteinized granulosa cells were treated with 200 nM rapamycin and 10 mM 3-MA and 200 μM H_2_O_2_ for 24 h. Rapamycin significantly increased the protein levels of Beclin1, Atg5, Lc3B II/I, Atg12, and Atg16L in H_2_O_2_-induced luteinized granulosa cell dysfunction, while 3-MA significantly decreased the protein levels of these autophagy-related genes (Figure 5A–F). The mRNA expression of *Beclin1*, *Atg5*, *Lc3B*, *Atg12*, and *Atg16L* was also upregulated by rapamycin and downregulated by 3-MA in H_2_O_2_-induced luteinized granulosa cells (Figure 5G–K).

These results demonstrated that rapamycin and 3-MA regulated autophagy, which could be further responsible for the regulation of H_2_O_2_-induced luteinized granulosa cell dysfunction.

### 2.6. 3-MA Inhibited the Activation of Autophagy Induced by Resveratrol

**Figure 6:**
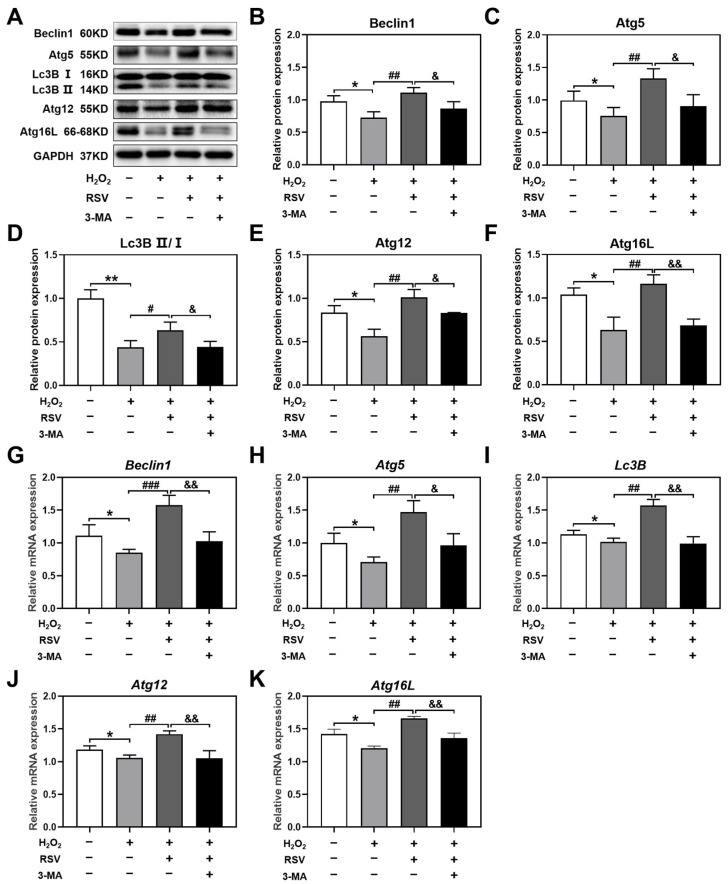
3-MA inhibited the activation of autophagy induced by resveratrol. Luteinized granulosa cells were treated with 20 μM resveratrol and 10 mM 3-MA and 200 μM H_2_O_2_ for 24 h. (**A**–**F**) The protein expression levels of Beclin1, Atg5, Lc3B II/I, Atg12, and Atg16L were detected using Western blotting and normalized to GAPDH (*n* = 3). (**G**–**K**) The mRNA expression levels of *Beclin1*, *Atg5*, *Lc3B*, *Atg12*, and *Atg16L* were detected using real-time PCR (*n* = 3). Compared with the control group, * *p* < 0.05, ** *p* < 0.01; compared with the H_2_O_2_ group, # *p* < 0.05, ## *p* < 0.01, ### *p* < 0.001; compared with the H_2_O_2_ + RSV group, & *p* < 0.05, && *p* < 0.01.

To further determine whether resveratrol activated autophagy by regulating the protein and mRNA levels of autophagy-related genes, luteinized granulosa cells were treated with the autophagy inhibitor 3-MA, and the levels of Beclin1, Atg5, Lc3B, Atg12, and Atg16L were detected using Western blotting and real-time PCR. Resveratrol significantly increased the protein and mRNA levels of Beclin1, Atg5, Lc3B, Atg12, and Atg16L in the H_2_O_2_ + RSV group compared with the H_2_O_2_ group. However, the administration of 3-MA attenuated the activation of these autophagy-related factors induced by resveratrol (Figure 6A–K). These results additionally supported that the increased levels of autophagy-related factors played an important role in the activation of autophagy induced by resveratrol, which could be reversed by 3-MA.

### 2.7. Resveratrol Controlled Autophagosome-Lysosome Fusion

**Figure 7:**
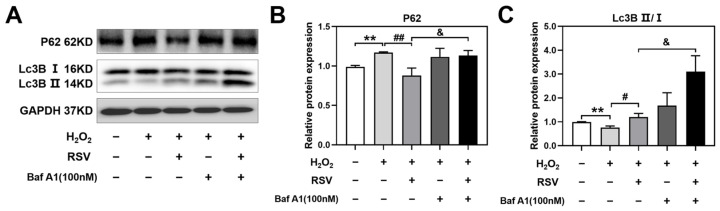
Resveratrol controlled autophagosome-lysosome fusion. (**A**–**C**) The protein levels of P62 and Lc3B II/I were detected using Western blotting and normalized to GAPDH (*n* = 3). Compared with the control group, ** *p* < 0.01; compared with the H_2_O_2_ group, # *p* < 0.05, ## *p* < 0.01; compared with the H_2_O_2_ + RSV group, & *p* < 0.05.

To determine whether resveratrol activated late autophagy and controlled autophagosome-lysosome fusion to eliminate extraneous or defective parts, bafilomycin A1 (Baf A1), an inhibitor of lysosomal degradation, was used to block late autophagy, and the autophagy markers P62 and Lc3B II/I were assessed by Western blotting. Luteinized granulosa cells were treated with 100 nM Baf A1 with or without 200 μM H_2_O_2_ and 20 μM resveratrol, and the dose of Baf A1 was based on a previous study [24]. Compared with the H_2_O_2_ + RSV group, the levels of P62 (*p* < 0.05) and Lc3B II/I (*p* < 0.05) were significantly increased in the H_2_O_2_ + RSV + Baf A1 group, indicating that enhanced autophagy induced by resveratrol could be further stimulated by Baf A1 (Figure 7A–C). These results illustrated that the administration of Baf A1 blocked the metabolism process of late autophagy, accumulated the levels of P62 and Lc3B II regulated by resveratrol, and finally confirmed the autophagosome-lysosome fusion activated by resveratrol.

## 3. Discussion

In this study, we found that resveratrol activated protective autophagy by upregulating the levels of autophagy-related genes to antagonize luteinized granulosa cell dysfunction. The increase in the protein and mRNA levels of Beclin1, Atg5, Lc3B, Atg12, and Atg16L involved in autophagy at least partially underlies the protective effect of resveratrol on luteinized granulosa cell dysfunction. These findings indicate a potential clinical application of resveratrol in the prevention and reversal of luteal dysfunction in vitro in H_2_O_2_-induced luteinized granulosa cells. However, the applications and involved molecular mechanisms of resveratrol in more female reproductive system diseases and more in vitro and in vivo models need to be further explored.

After ovulation, luteinizing hormone and follicle-stimulating hormone act on remnant follicles to stimulate the formation of the corpus luteum, entering the ovarian luteal phase. In this period, luteinized granulosa cells secrete progesterone and estradiol, becoming an important model in vitro to study the functions of the corpus luteum [25]. Twenty-one-day-old female presexual SD rats were treated with a combined hormone treatment of PMSG and hCG to induce ovarian luteinization, and the luteinized granulosa cells were successfully isolated and cultured in vitro according to the methods in some previous studies [26,27]. Although some red blood cells may mix with the luteinized granulosa cells, since the red blood cells cultured in suspension can be effectively removed by refreshing medium, it will not affect the subsequent experiments.

Our previous studies have reported decreased cell viability, damaged morphology and reduced secretion of estradiol in H_2_O_2_-induced mouse hippocampus-derived neuronal HT22 cells and rat ovarian granulosa cells [28,29]. In this study, we found that 200 µM H_2_O_2_ significantly decreased cell viability, inhibited the secretion of progesterone and estradiol, and increased the level of apoptosis in luteinized granulosa cells. Other researchers have also chosen H_2_O_2_ to induce oxidative dysfunction and have observed similar phenotypes in vitro [30,31]. Based on these results, we suggested that H_2_O_2_ effectively induced luteinized granulosa cell dysfunction.

Resveratrol has been reported to have a wide range of protective regulatory effects in many in vivo and in vitro studies. Lee et al. described the synergistic inhibitory effects of resveratrol and docetaxel on prostate carcinoma LNCaP cells, which were demonstrated by an increase in mitochondrial dysfunction, DNA damage response, and concurrent activation of apoptosis and necroptosis [32]. By suppressing the levels of TNF-α, IL-1β, and IL-6 in human retinal pigment epithelium cells, resveratrol inhibits inflammatory effects to ameliorate myopia development [33]. Deckmann et al. and Huang et al. also reported neuroprotection and cardioprotection of resveratrol in an autism animal model and a heart defect model, respectively [34,35]. In this study, 20 μM resveratrol treatment was proven to improve relative cellular viability, enhance the secretion of progesterone and estradiol, and alleviate apoptosis. Zhou et al. reported that resveratrol accelerated wound healing by attenuating H_2_O_2_-induced impairment of cell proliferation and migration [36]. Beatriz et al. confirmed an increase in estradiol production treated with resveratrol in the COV434 cell line and human granulosa cells [37]. Qin et al. revealed that resveratrol improved radiation-induced cell apoptosis by upregulating antioxidant enzymes and downregulating p53 acetylation [38]. These results are consistent with our findings and further support our hypothesis that resveratrol protects against H_2_O_2_-induced luteinized granulosa cell dysfunction.

Autophagy is required for cell homeostasis, differentiation, and survival by scavenging misfolded proteins, senescent or damaged organelles, and other intracellular toxic substances. Autophagy is involved in many important physiological processes, such as development, aging, and programmed cell death and plays a very important role in the pathogenesis of various diseases, such as cancer, cardiovascular diseases, and neurodegenerative diseases [39]. Some studies have noted that resveratrol stimulates autophagy activation to attenuate cell injury in osteoblasts in osteoporosis rats and human umbilical vein endothelial cells [40,41]. Hence, we further evaluated whether resveratrol regulates autophagy to resist cell dysfunction by observing the cellular ultrastructure. Autophagosomes derived from the wrapping of substrate organelles by phagophores and autophagolysosomes formed by the combination of autophagosomes and lysosomes are significant morphological markers representing autophagy [42]. In this study, increased formation of autophagosomes and autophagolysosomes was observed in the H_2_O_2_ + RSV group, indicating that autophagy can be activated by resveratrol and is involved in the protective effect of resveratrol against H_2_O_2_-induced luteinized granulosa cell dysfunction.

Furthermore, we investigated the molecular mechanism of autophagy activation by resveratrol and detected the expression levels of some key autophagy genes, such as Beclin1, Atg5, Lc3B, Atg12, and Atg16L. Beclin1 mediates the localization of other autophagy-related proteins to phagophores and regulates the formation and maturation of mammalian autophagosomes [43]. Atg5, Atg12, and Atg16L participate in the formation of the Atg5-Atg12-Atg16L conjugation system, which is necessary for autophagosome formation, phagophore elongation, and cargo recognition [44,45]. Lc3B is a marker protein located on the membrane of autophagosomes with two forms, Lc3B I and Lc3B II, and the conversion from Lc3B I to Lc3B II has been considered a specific marker of autophagy [46]. In this study, we found that the protein and mRNA levels of Beclin1, Atg5, Lc3B, Atg12, and Atg16L were significantly increased in the H_2_O_2_ + RSV group, suggesting that resveratrol upregulates autophagy-related genes to activate autophagy at the transcriptional and translational levels against H_2_O_2_-induced luteinized granulosa cell dysfunction.

Although Shao et al. reported the positive function of autophagy by regulating the differentiation of ovarian granulosa cells, activation of autophagy induced by bisphenol A has also been revealed to abnormally influence human ovarian functions and lead to abnormal folliculogenesis. In particular, few studies have focused on the role of autophagy in the regulation of corpus luteum function [47,48]. Our previous results revealed that the autophagy activated by resveratrol is closely related to its protection against H_2_O_2_-induced luteinized granulosa cell dysfunction. Thus, luteinized granulosa cells were treated with the autophagy agonist rapamycin and autophagy inhibitor 3-MA in the presence or absence of H_2_O_2_ to identify the protective effect of autophagy. Rapamycin, a specific inhibitor of the mammalian target of rapamycin complex 1 (mTORC1), has been well-reported to activate autophagy under a variety of physiological and pathological conditions [49,50]. Some studies reported that 100 nM and 200 nM rapamycin could activate autophagy to regulate apoptosis and cell viability in IL-18-induced chondrocytes and induced pluripotent stem cells [51,52]. In this study, we found that only 400 nM rapamycin significantly affected relative proliferation and viability in the absence of H_2_O_2_, while rapamycin showed a dose-dependent protective effect in the presence of H_2_O_2_. 3-MA can inhibit class III phosphatidylinositol-3-kinase (PI3K) and is widely used as an inhibitor of autophagy [53,54]. We found that 2.5~10 mM 3-MA induced significant relative proliferation and viability injury in the absence of H_2_O_2_, but only 10 mM 3-MA treatment aggravated cell injury in the presence of H_2_O_2_. These results indicate that inhibition of autophagy is more likely to cause dysfunction under physiological conditions in luteinized granulosa cells, while activation of autophagy achieved a sensitive protective effect in H_2_O_2_-induced luteinized granulosa cell dysfunction. Cotreatment of rapamycin and 3-MA with resveratrol further revealed the protective effects of autophagy on cell proliferation and activity and cell apoptosis in H_2_O_2_-induced luteinized granulosa cell dysfunction. Some supporting studies about the regulation of autophagy affecting endothelial apoptosis induced by cigarette smoke and inhibition of autophagy by 3-MA reversing the protective effects of resveratrol on podocytes also support this opinion [55,56].

**Figure 8:**
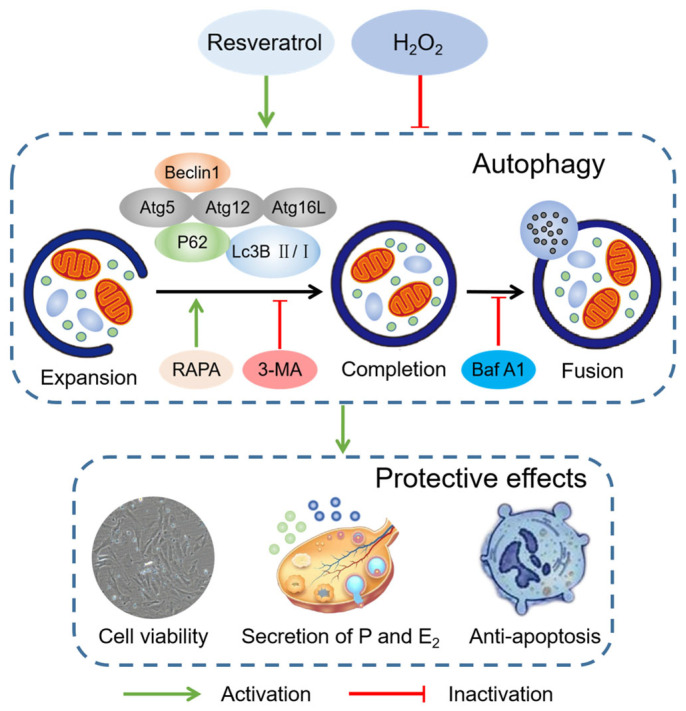
Mechanistic pathway of the protective effects of resveratrol on autophagy. RAPA, rapamycin; 3-MA, 3-methyladenine; Baf A1, bafilomycin A1; P, progesterone; E_2_, estradiol.

Additionally, the protein and mRNA levels of autophagy-related genes were detected to evaluate the regulation of autophagy by rapamycin and 3-MA. In this study, we found that rapamycin activated the expression levels of autophagy-related genes and that 3-MA inhibited them, which further confirmed the correlation of autophagy and dysfunction regulated by rapamycin and 3-MA in H_2_O_2_-induced luteinized granulosa cell dysfunction. We found that the administration of 3-MA inhibited the activation of autophagy-related genes by resveratrol. Resveratrol has been reported to activate autophagy via the PI3K-AKT pathway against disc degeneration and spinal cord dysfunction, which can be reversed by 3-MA [57,58]. Finally, luteinized granulosa cells were treated with bafilomycin A1, an inhibitor of late autophagy, to evaluate the regulatory effect of resveratrol on autophagosome-lysosome fusion. The accumulation of the cargo protein P62 and autophagosomal structure protein Lc3B II induced by bafilomycin A1 indicated that resveratrol activated autophagy. The effects of bafilomycin A1 on P62 and Lc3B II in the regulation of autophagosome-lysosome fusion and degradation have been widely verified in some studies of melatonin resistance to abdominal aortic aneurysm and quercetin interference with osteosarcoma cells [59,60]. These results further support our hypothesis that resveratrol activates autophagy by regulating the protein and mRNA levels of autophagy-related genes, thereby protecting against H_2_O_2_-induced luteinized granulosa cell dysfunction in terms of enhancing cell viability, improving the secretion of progesterone and estradiol, and inhibiting apoptosis (Figure 8).

## 4. Materials and Methods

### 4.1. Animals

Twenty-one-day-old female SD rats were subcutaneously injected with 50 IU pregnant mare serum gonadotropin (PMSG) to promote gonad development, and after 48 h, they were intraperitoneally injected with 50 IU human chorionic gonadotrophin (hCG) to induce ovarian luteinization. After 7 days, the ovaries were collected for the isolation of rat ovarian luteinized granulosa cells. The rats were purchased from the animal experiment center of Harbin Medical University (approval code: QC2017089), and before sacrificing, they were housed with free access to standard rat chow and tap water under room temperature and natural light.

### 4.2. Rat Ovarian Luteinized Granulosa Cell Isolation and Culture

Luteinized ovaries stripped of the Fallopian tube, fat, and connective tissue were washed with PBS and cut into a paste. Luteal tissue was mixed with 0.25% collagenase II (Gibco, Anaheim, CA, USA) and digested with shaking for 30 min at 37 °C, and then, luteinized granulosa cells were purified by filtration with a 200-μm stainless steel mesh. After centrifugation at 500× *g* for 10 min, the cells were resuspended in DMEM/F12 medium supplemented with 10% FBS and 1% penicillin/streptomycin and then cultured in a 5% CO_2_ incubator at 37 °C.

### 4.3. CCK-8 Assay

Relative cellular viability was measured using the CCK-8 assay. Luteinized granulosa cells were seeded at a density of 5 × 10^4^ cells in each well of 96-well plates and treated with different reagents as needed for 24 h. The reagent information is shown as follows: hydrogen peroxide (Hushi, Shanghai, China), resveratrol (Aladdin, Shanghai, China), rapamycin (Aladdin, Shanghai, China), 3-methyladenine (Aladdin, Shanghai, China), and bafilomycin A1 (GLPBIO., Shanghai, China). The cells were further incubated in 200 μL DMEM/F12 medium supplemented with 20 μL of CCK-8 reagent (Yiyuan Biotechnology, Guangzhou, China) for 2 h. The OD value was measured at a wavelength of 450 nm, and the relative cellular viability was normalized to the value of the control group.

### 4.4. Hormone Detection

The levels of progesterone and estradiol in the culture supernatant of the luteinized granulosa cells were detected using commercial radioimmunoassay kits (Sino-UK Institute of Biological Technology, Beijing, China).

### 4.5. JC-1 Staining

JC-1 is a membrane-permeable cationic dye used to study mitochondrial integrity in early cell apoptosis. JC-1 can selectively enter mitochondria and change its fluorescence properties with changes in mitochondrial membrane potential. The level of red and green fluorescence stimulated by JC-1 can be used to determine early apoptosis. Luteinized granulosa cells were seeded at a density of 2 × 10^5^ cells in each well of 24-well plates and treated with different reagents as needed for 24 h. According to the instructions of the JC-1 staining kit (Elabscience, Wuhan, China), the cells were dyed with working solution for 20 min and washed with JC-1 buffer 2 times. Images were acquired with a Nikon E800 fluorescence microscope and quantitatively analyzed by ImageJ software version 1.46r.

### 4.6. Observation of Autophagy Structure

The formation of autophagolysosomes and autophagosomes was observed by transmission electron microscopy. The luteinized granulosa cells were seeded at a density of 2.5 × 10^6^ cells in T-25 cell culture flasks and were divided into the control group, the H_2_O_2_ group, and the H_2_O_2_ + RSV group. The luteinized granulosa cells were digested by 0.25% trypsin and collected after centrifugation at 800× *g* for 10 min. Then, 500 μL of glutaraldehyde was added to the luteinized granulosa cells, and the samples were sent to the electron microscope center of Harbin Medical University for transmission electron microscopy observation.

### 4.7. Western Blot

The protein levels of the autophagy-related genes were determined by Western blot analysis. Briefly, 15 μg of total proteins extracted from the luteinized granulosa cells were electrophoresed on 10% or 12% SDS-PAGE gels and transferred onto PVDF membranes. After blocking, the membranes were washed with TBST and incubated with primary antibodies at 4 °C overnight. GAPDH (1:3000), Bcl-2 (1:1000), Bax (1:1000), P62 (1:1000) were purchased from Affinity Biosciences and Beclin1 (1:1000), Atg5 (1:1000), Lc3B (1:1000), Atg12 (1:1000), Atg16L (1:1000) were purchased from Cell Signaling Technology. The following day, the membranes were washed with TBST and incubated with horseradish peroxidase (HRP)-conjugated goat anti-rabbit IgG antibody at room temperature for 1 h. The bound antibody was visualized with an enhanced chemiluminescent detection kit (Engreen Biosystem, Beijing, China) and captured under a chemiluminescence imaging system (Tanon, Shanghai, China). The relative densities of the bands normalized by GAPDH, which was reported as a proper loading control than actin and tubulin were assessed by ImageJ software version 1.46r [61].

### 4.8. Real-Time PCR

The mRNA levels of the autophagy-related genes were determined by real-time PCR. Briefly, Total RNA was extracted from the luteinized granulosa cells using TRIzol (Ambion, Austin, TX, USA) and used in the synthesis of cDNA by a PrimeScript 1st Strand cDNA Synthesis Kit (Takara, Tokyo, Japan). The real-time PCRs were performed using TB Green^®^ Premix Ex Taq™ II Kit (Takara, Tokyo, Japan) in Applied Biosystems 7500 Real-Time PCR System. The relative mRNA expression normalized by β-actin was quantitatively calculated by the ΔΔCt method, which was referred to in a previous study [62]. The sequences of the primers used in real-time PCR are listed in Table 1.

### 4.9. Statistical Analysis

Data were presented as mean ± standard deviation. Statistical differences between groups were calculated by one-way ANOVA using the GraphPad Prism 8 software. *p* < 0.05 was considered to indicate a statistically significant difference.

## 5. Conclusions

In summary, this study focused on the role of resveratrol in protecting against luteal dysfunction and revealed the molecular mechanisms of protective autophagy activated by resveratrol in vitro. Resveratrol alleviated H_2_O_2_-induced luteinized granulosa cell dysfunction by increasing cell viability, stimulating the secretion of progesterone and estradiol, and ameliorating cell apoptosis. Increased formation of autophagosomes and autophagolysosomes induced by resveratrol prompted the correlation between this protective effect and the activation of autophagy. Resveratrol activated autophagy-related genes such as Beclin1, Atg5, Lc3B, Atg12, and Atg16L at the transcriptional and translational levels. Treatment with rapamycin, 3-MA, and bafilomycin A1 further confirmed the protective role of autophagy. These results indicate that resveratrol activates autophagy to resist H_2_O_2_-induced oxidative dysfunction by upregulating the expression of autophagy-related genes, providing a deeper understanding of resveratrol’s protective and antioxidant activities in the ovary.

## Figures and Tables

**Table 1:** Sequences of the primers used in real-time PCR.

Gene	Type	Sequences (5′ → 3′)
*Beclin1*	Forward Primer	GCCTCTGAAACTGGACACG
*Beclin1*	Reverse Primer	CCTCTTCCTCCTGGCTCTCT
*Atg5*	Forward Primer	CACTGGGACTTCTGCTCCTG
*Atg5*	Reverse Primer	TTCTTCAACCAAGCCAAACC
*Lc3B*	Forward Primer	GGTGTTTTTCTCCTGGTTTGG
*Lc3B*	Reverse Primer	GCACTTGGACTTCAGCCTTC
*Atg12*	Forward Primer	AAACGAAGAAATGGGCTGTG
*Atg12*	Reverse Primer	GAAGGGGCAAAGGACTGATT
*Atg16L*	Forward Primer	CTGTGCTTTTCCCGTCTTTC
*Atg16L*	Reverse Primer	GCCCTGATTTGGTTTCCAC
*β-actin*	Forward Primer	AGCCATGTACGTAGCCATCC
*β-actin*	Reverse Primer	GCTGTGGTGGTGAAGCTGTA

## Data Availability

Not applicable.
